# Randomized, single‐centre, double‐blinded repeated dose investigation of safety and tolerability of ocular administrations of a novel silica eye drop product in healthy volunteers

**DOI:** 10.1111/aos.70061

**Published:** 2026-02-02

**Authors:** Aleksandra Poluianova, Marceline N. Akieh‐Pirkanniemi, Arto Hartikainen, Hanna Arstila, Kai Kaarniranta, Lasse Leino

**Affiliations:** ^1^ Helsinki Eye Lab Helsinki University Eye Hospital Helsinki Finland; ^2^ Department of Ophthalmology University of Eastern Finland Kuopio Finland; ^3^ DelSiTech Ltd. Turku Finland; ^4^ Department of Ophthalmology Kuopio University Hospital and University of Eastern Finland Kuopio Finland; ^5^ Department of Life Technologies University of Turku Turku Finland

**Keywords:** clinical trial, drug carrier, eye drops, ophthalmology, silica gel, silica matrix, tolerability

## Abstract

**Purpose:**

Managing ocular diseases often requires frequent eye drop administration, which can challenge patient compliance. A long‐acting eye drop technology using an amorphous synthetic silica composite was developed to address this. Our study aimed to assess the safety and tolerability of the Silica Eye Drop platform in healthy volunteers over 15 days.

**Methods:**

Twelve healthy volunteers participated in a randomized, double‐blinded, placebo‐controlled trial of the Silica Eye Drop Product, containing no active substance. Participants received one drop in one eye daily for 13 days, with the other eye serving as an untreated control. Safety and tolerability were evaluated through various examinations at multiple time points. Ocular discomfort was assessed with a questionnaire at these times, and additional evaluations of lens, vitreous body, visual acuity (BCVA), ocular protection index (OPI), and intraocular pressure (IOP) were performed at t0 and D15.

**Results:**

No significant differences in ocular metrics between treated and untreated eyes were observed after 15 days of Silica Eye Drop application (IOP: control 14.1 ± 2.02 mmHg, treated 13.6 ± 1.75 mmHg; BCVA: control 1.22 ± 0.16, treated 1.24 ± 0.15; OPI: control 1.66 ± 0.43, treated 1.63 ± 0.40). Questionnaire responses indicated that 68% of volunteers experienced mild discomfort during the product application, while 32% noted moderate discomfort. The average pleasantness score was 4.9 ± 1.83 using a 10‐point scale, indicating acceptable tolerability of Silica Eye Drops.

**Conclusions:**

The findings suggest that Silica Eye Drops are safe and well‐tolerated by study subjects when used once daily. This supports further developing sustained release topical ocular products for delivering pharmacological treatments in various eye conditions.

## 1 INTRODUCTION

Topical administration of drugs for ophthalmic conditions often requires frequent dosing, usually multiple times per day, due to the rapid clearance of the drug from the ocular surface. This can be attributed to several factors, including the complex anatomical structure of the eye, the small porous surface area, the low permeability of the cornea, the lipophilicity of the corneal epithelium, metabolic processes, enzymatic degradation, the binding of the drug to proteins in the tear fluid, and protective mechanisms such as tear production, blinking and drainage through the nasolacrimal duct (Gote et al., 2019). Due to the small volume of the conjunctival sac, approximately 30 μL without blinking, and the presence of protective mechanisms, the concentration of the drug at the application site decreases, leading to a reduction in the duration of action of the active substance (Forrester et al., 2016). Additionally, a significant issue in ophthalmology regarding drug usage is patient compliance. Many eye drop medications contain preservatives and other additives that cause discomfort, burning, and stinging, leading to decreased adherence among various patient groups (Amiri et al., 2024; Fineide et al., 2024; Hedengran et al., 2022; Hynnekleiv et al., 2024; Iribarren et al., 2024; Moore et al., 2023; Nagstrup, 2023; Utheim et al., 2025). Furthermore, it has been demonstrated that patient compliance improves when the frequency of eye drop administration is reduced, indicating that patients are less likely to overlook their treatment regimen (Hasebe et al., 2018). Thus, there is a definite need to maintain the concentration of the drug at the site of action for a longer period, which will contribute to a lower frequency of administration and increased bioavailability, as well as increased efficacy of the drug. Recently, prodrugs, cyclodextrins, in situ gels, and nanoparticles have been studied to improve ophthalmic bioavailability (Stefansson et al., 2023; Wang & Wang, 2022).

Amorphous, synthetic silica (silicon dioxide, SiO_2_) composite materials, consisting of silica microparticles embedded in silica hydrogel, have been investigated as carriers for controlled and sustained delivery of active substances (Forsback et al., 2021; McGowan et al., 2024; Noppari et al., 2020; Tyagi et al., 2018, 2022). The controlled biodegradation rate and nanoscale porous structure of silica microparticles, combined with the mild and aqueous processing conditions of the composite components, make it an ideal material for controlled drug delivery. The silica carrier matrix can incorporate various active substances, such as antibacterial, antiviral, anti‐inflammatory, ocular pressure‐reducing, and other pharmacologically active substances. The application of this technology in ophthalmic drug delivery holds significant promise for the future.

In contact with body fluids, the silica composite matrix dissolves, and the encapsulated active substance (i.e., cargo) is released gradually. The silica matrix dissolution occurs mainly through a surface erosion mechanism (Rosenholm et al., 2010). Eroded silica is freely dispersed in tissue and excreted via urine as a weak inorganic silicic acid. The biodegradation process unequivocally does not impact the pH within the silica gel or surrounding tissue. By adjusting the manufacturing process, it is possible to vary the number of hydroxyl groups and specific surface area (from dense to highly porous) of silica microparticles, which both have an influence on the biodegradation rate of silica and hence the release rate of the active substance (Jokinen et al., 2008; Viitala, Jokinen, Maunu, et al., 2005; Viitala, Jokinen, Tuusa, et al., 2005).

The safety and tolerability of silica composite formulations have been extensively studied using multiple routes of administration (subcutaneous, intradermal, intra‐articular, intramuscular, intracameral, intravitreal, topical to the eye) in various animal models (Hirai et al., 2012; Jin et al., 2020; Kim & Lee, 2023; Leclerc et al., 2015; Liao et al., 2017; Sun et al., 2020; Sun et al., 2021). These studies have consistently demonstrated that silica composite formulations are well tolerated in animals.

In this article we report for the first time the safety, tolerability, and clinical acceptability of the silica composite matrix in human subjects after repeated topical administration in the eye. An experimental Silica Eye Drop Product, consisting of a silica composite matrix made of silica microparticles and silica hydrogel, was investigated as a representative formulation for the technology platform for long‐lasting delivery of active substances in the front of the eye.

## 2 MATERIALS AND METHODS

The clinical study was carried out in line with the ISO 14155:2020, Good Clinical Practice (GCP) guidelines and the ethical standards of the Declaration of Helsinki. The study protocol was approved by the Ethics Committee of the North Savo Hospital District and FIMEA (Finnish Medicines Agency). As a medical device study conducted in Finland, it was not required to include it in the EU Clinical Trials Register. Before any screening procedures, all volunteers provided their written informed consent. All data entry, verification, data validation, medical encoding and quality control activities with acceptable error rates were finalized before the final database lock (hard lock).

### 2.1 Study design

**FIGURE 1:**
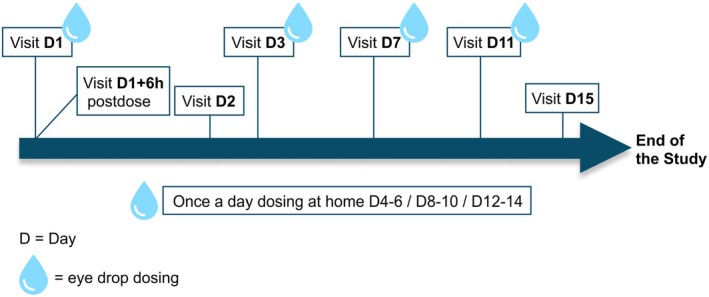
Schematic of the study design. The clinical study began with an initial visit to the clinic, where a physical examination was conducted, and IMD was instilled in one eye. After 6 h, a clinical assessment was performed. Subjects returned to the clinic the next day for repeat studies. They returned to the clinic on Days 3, 7 and 11 for IMD instillation and new assessments. Volunteers spent Days 4–6, 8–10 and 12–14 at home, administering the Silica Eye Drop themselves. On the last Day 15, they arrived at the clinic for final examinations without having instilled IMD.

This was a first‐in‐human, single‐centre, randomized, double‐blinded, repeated‐dose investigation in healthy volunteers. It was considered a pilot‐stage, exploratory medical device clinical investigation. The study evaluated the safety and tolerability of repeated ocular administrations of the Silica Eye Drop Product (the Investigational Medical Device, IMD). There were no efficacy endpoints in this phase because the IMD did not contain any active substance. The study participants received one drop in one eye daily for 13 days during a two‐week period (Figure 1), while the other eye acted as a control and did not receive any placebo treatment. The decision regarding which eye received the treatment and which served as the control was determined by a randomization list during the screening and baseline visit on Day 1 of the study. The randomization took place after eligibility was confirmed through an ophthalmological examination. The Investigator was not informed about which eye received the treatment. Nonetheless, the drop administration was carried out by a separate, unblinded study nurse at the hospital on check‐up days or by the participant themselves at home.

### 2.2 Silica eye drop product

The Silica Eye Drop Product is composed of a silica‐silica composite material containing silica microparticles embedded in a silica hydrogel and packaged in polyethylene single‐dose units. The synthetic route of silica followed the well‐known chemical reaction called the sol‐gel process. In the sol‐gel process, colloidal silica, that is a suspension of small spherical and amorphous silica nanoclusters, was produced by hydrolysis of a precursor molecule, tetraethyl orthosilicate (TEOS, Sigma‐Aldrich), in an acid‐catalysed environment. In the hydrolytic reaction, the ethoxy side chains of TEOS were hydrolysed, and ethanol was formed as a by‐product. The colloidal silica suspension (silica sol) was spray‐dried to produce silica microparticles with a typical average diameter (D50 value) of approximately 5 μm. The silica content of the microparticles was 74 weight%.

**FIGURE 2:**
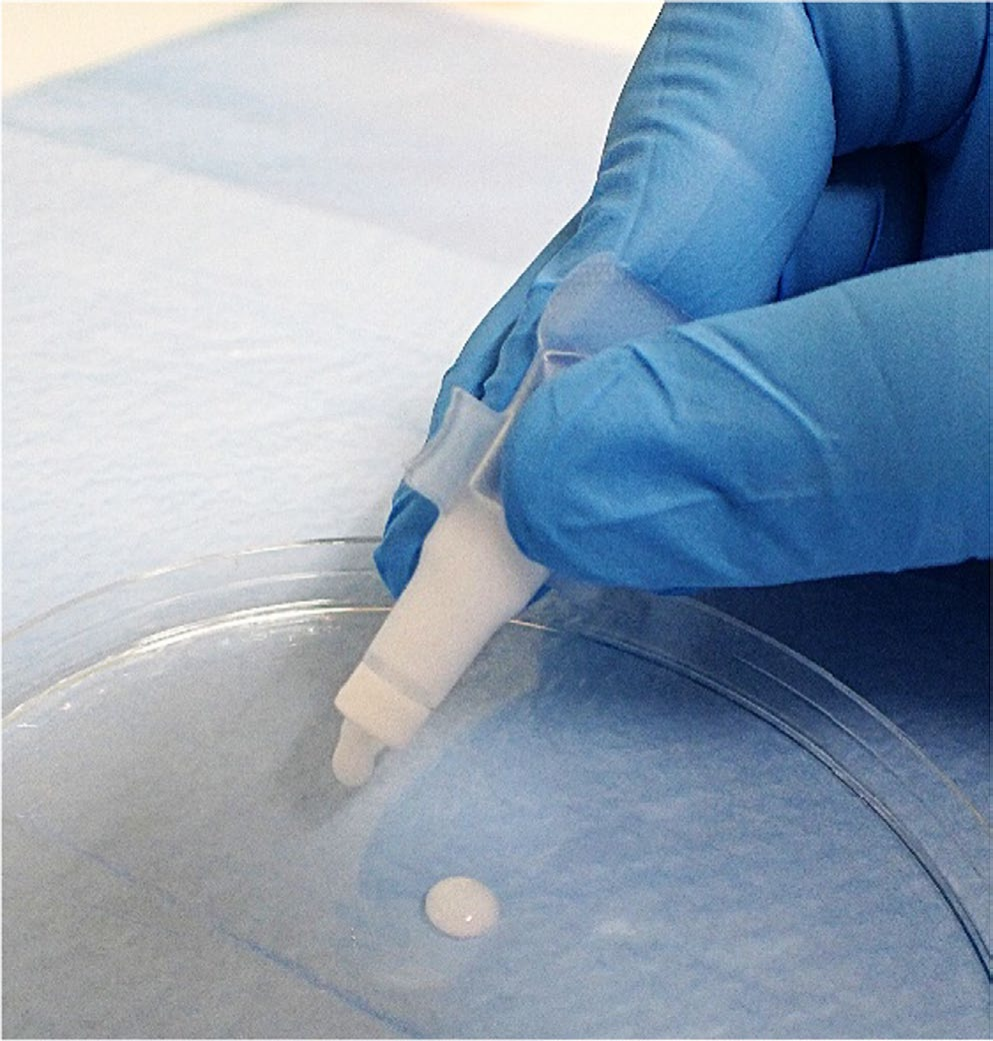
A photo of the silica eye drop product in the SDU primary package. A drop of approximately 30 μL was applied topically in the cul‐de‐sac by gently squeezing the SDU.

After the spray drying process, the silica microparticles were mixed with a dilute silica sol (0.3 g microparticles in 1 m of sol) to form a white suspension that turned into a hydrogel after stabilization at room temperature for a day. Prior to the stabilization phase, the suspension was transferred into polyethylene single‐dose units (SDUs) from Lameplast with a fill volume of 0.8 mL per SDU. One drop of Silica Eye Drop was approximately 30 μL. The SDUs were packed in aluminium pouches (from Daklapack) and stored at 5 ± 3°C before sterilization by gamma irradiation. The final products were gamma irradiated at 30 kGy, and sterility was demonstrated at the sterility assurance level (SAL) of 10^−6^. Figure 2 shows a photograph of the Silica Eye Drop in the SDU primary package. The Silica Eye Drop Product was approved for clinical use following a successful release analysis, which included assessment of appearance, assay for silica content, in vitro dissolution, residual solvents, pH, sterility, and endotoxin levels. The anticipated biodegradation time after topical application of the silica composite matrix was about 9 h based on an accelerated in vitro in‐sink dissolution test method described earlier (Tyagi et al., 2022).

The silica microparticles were manufactured under Good Manufacturing Practice (GMP). The fill‐and‐finish process was done according to the International Organisation for Standardisation (ISO) 13485.

### 2.3 Subjects

In this study, a total of 12 healthy adult volunteers (9 males and 3 females) were screened and included. The main inclusion criteria required volunteers to be healthy, 18–55 years of age, have an intraocular pressure (IOP) ≤21 mmHg with a difference of <4 mmHg between eyes, an ocular surface disease index (OSDI) score of ≤20, and have no history of significant eye disease or any current eye disease that could impact the assessment of safety and tolerability of the IMD. Volunteers were not eligible if they had used any eye medications in the last 3 months, including artificial or moisturizing tears, had an allergy to Tropicamide or Tafluprost, or had worn contact lenses or eyelash extensions in the previous 3 months. Furthermore, individuals with general sensitivity in their eyes (such as eye drops, wind, chlorine, smoke, fumes, scents, dust, pollen, or make‐up) and seasonal allergies were not included in the study. Sensitivity was assessed through self‐reports from each volunteer.

The subjects were instructed to keep their use of all medications, dietary supplements, and treatments the same as reported at the screening visit until the last visit. The use of artificial or moisturizing tears was restricted throughout the entire study. If a new concomitant medication was needed, the subject was to inform the investigator, and the investigator decided on a case‐by‐case basis whether to continue participation.

### 2.4 Assessments

The safety and tolerability of the IMD were evaluated through ophthalmological examinations and by recording the sensations reported by the subjects after exposure. The volunteers were given a structured questionnaire to score the maximum intensity and duration of ocular discomfort. The discomfort was examined in terms of, for example, pain or soreness, sensitivity to light, the sensation of a piece of sand or a foreign body in the eye after dosing the product. The subjects scored the discomfort level from 0 (no signs or symptoms) to 3 (severe), and the duration of discomfort was scored from 0 (no signs or symptoms) to 3 (1 h or more). The questionnaires were collected on the predefined visiting days (Day 1 + 6 h, Day 3, Day 7, Day 11, and Day 15), providing a total of 60 responses.

Physical assessment of the eyes was performed by an ophthalmologist before the first dose and on Days 1 (after 6 h of the application), 2, 3, 7, 11, and 15 (end of the study). The examination included combinations of the following tests: the best corrected visual acuity (ETDRS (Early Treatment Diabetic Retinopathy Study) table, logMAR scale), assessment of bulbar conjunctival and lid margin redness using Institute of Eye Research (IER) grading scales (Laihia et al., 2020; Schulze et al., 2009), corneal and conjunctival fluorescein staining patterns according to an Oxford grading scale, anterior chamber cells and flare and interblink interval and tear fluid break‐up time. The same assessments were performed on both eyes. Both the nasal and temporal parts of the conjunctivae were evaluated. In case the nasal and temporal staining patterns differed, the score to be recorded was the higher of the two. Intraocular pressure, lens evaluation, and examination of the vitreous body and eye fundi (macula, peripheral retina, and papilla) were performed on Days 1 (before the study) and 15 (end of the study). The pupil was dilated with Tropicamid 5 mg/mL (Santen Oy, Tampere) in both treated and control eyes prior to lens examinations.

The ocular protection index (OPI) was calculated for each participant for treated and untreated eyes at each time point. It is a significant parameter for assessing dry eye syndrome. OPI was calculated by dividing the Tear fluid breakup time (TBUT) by the Interblink interval (IBI) during 1 min. When the OPI is <1.0, the patient's ocular surface is exposed, putting them at risk for developing the signs and symptoms of dry eye. On the other hand, if the OPI is ≥1.0, the patient's ocular surface is protected by the tear film.

Clinical acceptability was evaluated by the participants' pleasantness of use. At the end of the study, the volunteers were asked to provide feedback on the pleasantness of the Silica Eye Drop using a 10‐point scale. The scale ‘0’ represents ‘very unpleasant’, and ‘10’ represents ‘very pleasant’.

### 2.5 Statistical methods and determination of sample size

The number of subjects included in this exploratory investigation was based on clinical considerations. No formal sample size calculation was performed.

The subjects' baseline characteristics were tabulated with descriptive statistics by treatment. A detailed statistical analysis plan was prepared prior to the database lock. No formal statistical hypotheses were pre‐defined for this study. Subjects who received at least one dose of the IMD and had any follow‐up data were considered evaluable for safety and tolerability. A subject classification document was prepared before the opening of the randomization code for final analysis.

Ocular signs (lid margin and bulbar conjunctival redness, conjunctival, and corneal fluorescein staining) were summarized descriptively (visual inspection) by treatment, time point, and eye. At each visit, treated eyes were compared to untreated eyes to evaluate the tolerability of the single‐dose and repeated‐dose Silica Eye Drop Product. To effectively compare the single‐dose and repeated‐dose of the IMD, measurements at D1 (after 6 h), D2, and D3 were compared to the measurements at D7, D11, and D15.

Statistical analysis was performed using GraphPad Prism software version 10.4.1 (532) (GraphPad Software, San Diego, CA, USA). Non‐parametric tests were employed to analyse IOP, best‐corrected visual acuity (BCVA), and OPI due to the small sample size and the non‐normal distribution of the data, as determined by the Shapiro‐Wilk test. Comparisons between independent groups (treated vs. control) were made using the Mann‐Whitney U test, and within‐group comparisons were performed using the Wilcoxon signed‐rank test. Statistical significance was defined as *p* < 0.05.

Tolerability was evaluated using an ocular discomfort questionnaire. Intensity and duration of discomfort were summarized descriptively using frequency tables at each time point. Adverse Events (AEs) were summarized by system organ class and preferred term using frequency tables. Ocular AEs and AEs related to the study treatment were tabulated.

## 3 RESULTS

All 12 enrolled and randomly selected participants successfully completed all stages of the study. The mean age of the included subjects was 28.3 years (range 18–46 years). The Silica Eye Drop Products were at least moderately tolerated and safe, with no serious AEs or other significant issues. Only two participants experienced AEs throughout the study, specifically myokymia and a dry sensation in the treated eye. However, after evaluation, the Investigator determined that these events were not related to the study treatment. Nevertheless, the severity of these events was reported as mild, and they did not require additional treatment.

**FIGURE 3:**
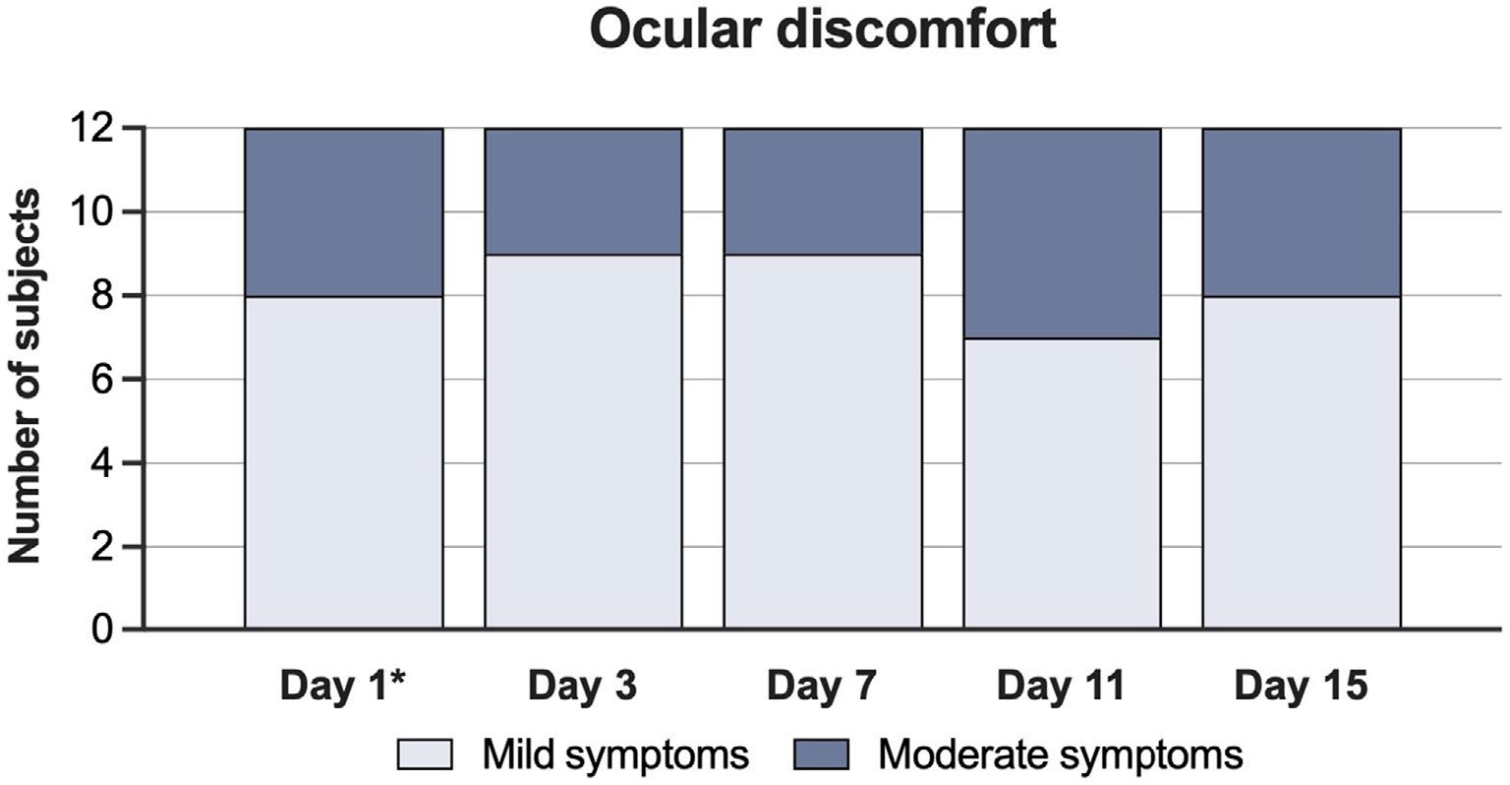
Ocular discomfort over time was assessed during visits in treated eyes. Data was collected using a subject questionnaire on predefined days. Day 1* means visit on the first day and 6 h after dosing.

At the beginning of the study, after 6 h of IMD application, 8 participants reported mild ocular discomfort, while 4 participants noted moderate ocular symptoms. After processing 60 questionnaires from volunteers, 68% experienced mild discomfort (score of 1), while 32% reported moderate discomfort (score of 2). None of the subjects indicated severe ocular discomfort. The individual subject results are shown in Figure 3. Among those who experienced discomfort, 96% reported it lasted less than a minute, 3% reported it lasted between 1 and 59 min and 1% reported it lasted an hour or more. The study showed no notable increase in symptoms, indicating that ocular comfort remained stable with the privilege of mild effects.

During clinical examinations, all 24 eyes (both control and treated) received the same score of ‘0 = very slight’ for lid margin and bulbar conjunctival redness at all time points. Corneal and conjunctival fluorescein staining patterns from subjects registered 1 (absent) and 2 (minimal), with 1 (absent) being the most common score. Three out of 12 participants were scored 2 (minimal) on different days: specifically, one participant on Day 3, another on Day 11, and a third on Day 15. This participant also scored 2 for the untreated control eye. No clinically meaningful changes were noted for any of the subjects.

Additionally, the examination of the anterior chamber, specifically focusing on the presence of cells and flare, did not reveal any pathological changes at any of the time points. Furthermore, following a 15‐day clinical trial, there were no unexpected safety observations or changes in lens transparency or fundus examination (papilla, macula, and peripheral retina). An abnormality of non‐clinical significance was observed in the vitreous body and eye fundi examination on Day 1 for 1 out of 12 subjects. This abnormality was also seen in the untreated eye.

**FIGURE 4:**
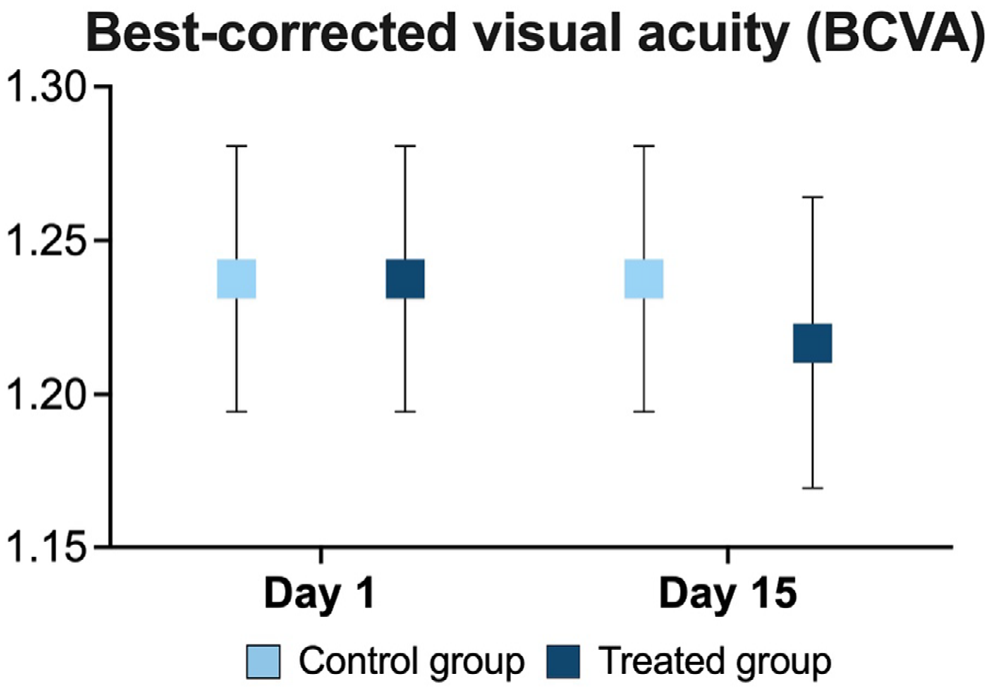
Mean BCVA scores are shown for baseline (Day 1) and Day 15 in both treated (*n* = 12) and control (*n* = 12) groups. Error bars represent the standard error of the mean (SEM).

BCVA was analysed in treated and untreated eyes on Day 1 and Day 15 using ETDRS in the sitting position. The baseline results were compared to the results obtained on Day 15 (Figure 4). There were no statistical differences in BCVA between the treated eye and the untreated eye at any time points (Day 1: *p* = 1.0, Day 15: *p* = 0.717) or between screening and end‐of‐study visits (control group: *p* = 1.0, treated group: *p* = 0.317).

**FIGURE 5:**
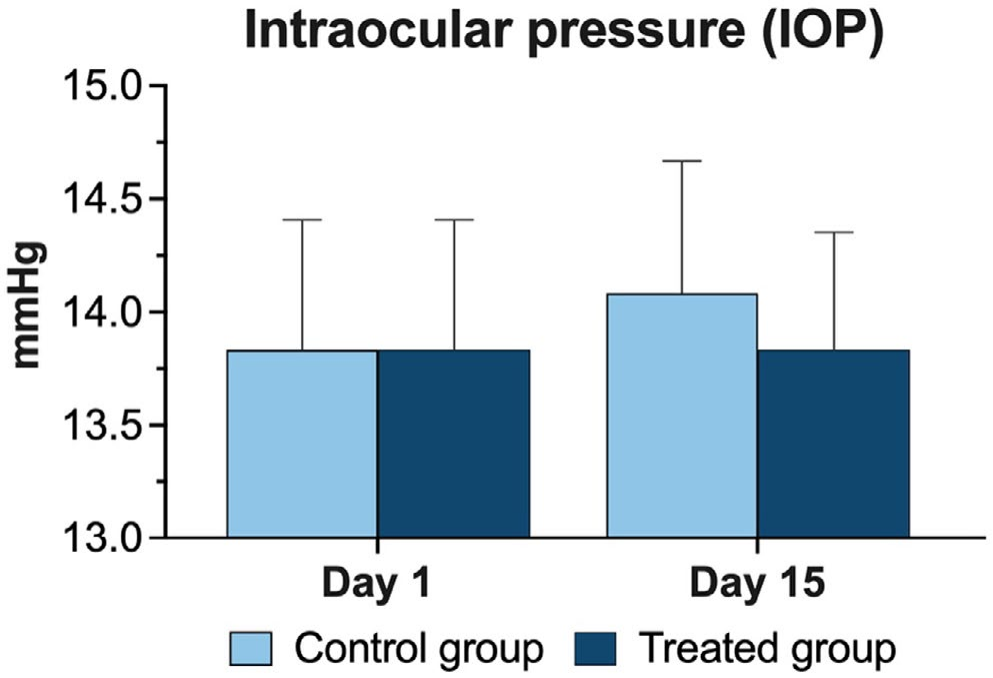
Mean IOP values are shown for baseline (Day 1) and Day 15 in both treated (*n* = 12) and control (*n* = 12) groups. Error bars represent the standard error of the mean (SEM).

Moreover, IOP was nearly the same after 15 days of study compared to the first day (Figure 5). There were no statistical changes in both the between‐group (Day 1: *p* = 1.0, Day 15: *p* = 0.736) and intergroup (control group: *p* = 0.414, treated group: *p* = 1.0) comparisons, indicating the safety of Silica Eye Drops regarding its impact on the circulation of intraocular fluid.

**FIGURE 6:**
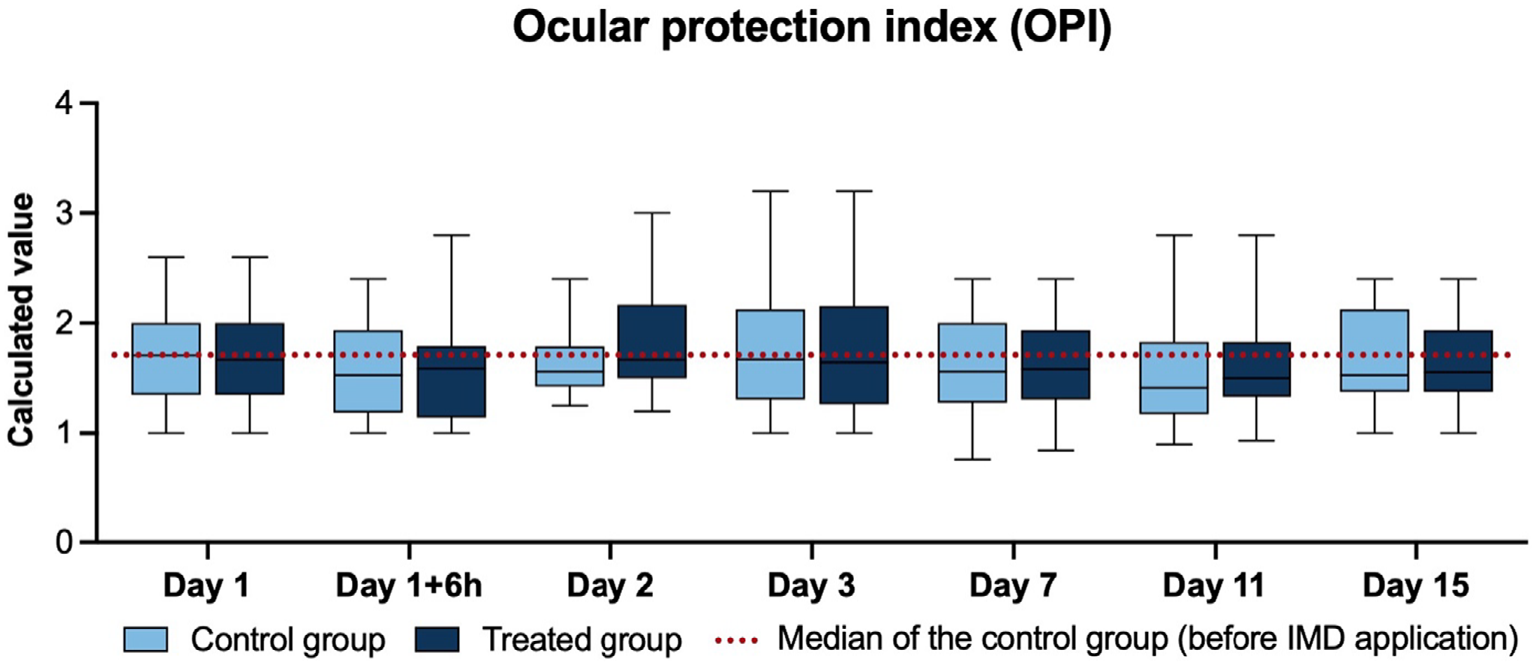
OPI values at each time point for the treated (*n* = 12) and control (*n* = 12) groups. Boxes represent the interquartile range (IQR), horizontal lines indicate the median and whiskers show the minimum and maximum values. The red dotted line represents the median OPI value of the control group before IMD application.

In addition, OPI was determined for every participant at each time point for both treated and untreated eyes. The change in OPI from baseline was also calculated for each visit, showing little to no change compared to the control group before IMD application (Figure 6). The average OPI was ≥1.0, suggesting that the tear film effectively protected the ocular surface and that using the product did not lead to the development of dry eye disease. Before Silica Eye Drop instillation on Day 1, the mean ± SD OPI was 1.74 ± 0.44 for the control group and 1.72 ± 0.47 for the treated group (*p* = 0.89), indicating no significant difference. On Day 2, the treated group displayed a slightly higher mean OPI (1.78 ± 0.48) compared to the control group (1.65 ± 0.34), though this difference was not statistically significant (*p* = 0.47). At later time points, OPI values remained comparable between groups. For instance, at Day 15, the mean OPI was 1.65 ± 0.43 in the control group and 1.63 ± 0.40 in the treated group (*p* = 0.87). Overall, no statistically significant differences in OPI were observed between treated and control groups at any time points (all *p* > 0.05).

**FIGURE 7:**
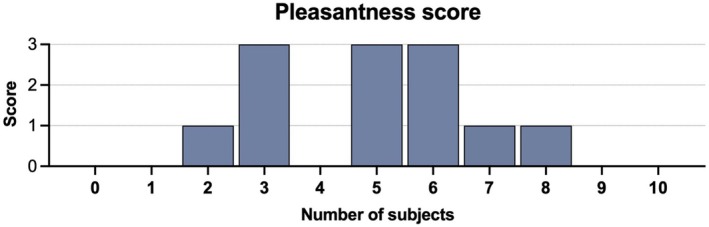
Overall pleasantness of use of the IMD. Each score corresponds to the number of subjects who provided the score.

Clinical acceptability was assessed at the end of the study, as illustrated in Figure 7. The highest score was 8, and the lowest score was 2. The median score was 5.5. The average score given by respondents was 4.9 ± 1.83, suggesting that IMD demonstrated a moderate level of tolerability during the testing phase.

## 4 DISCUSSION

The primary objective of the randomized, double‐blinded, and placebo‐controlled clinical trial was to obtain data on the safety and tolerability of the topical ocular Silica Eye Drop Product treatment in healthy volunteers. After administration, the Silica Eye Drop Product was well‐tolerated and deemed safe despite some mild to moderate symptoms reported. It is important to note that conventional medications are usually prescribed 2–4 times a day, while the Silica Eye Drop Product can be required once a day. This means that any mild symptoms associated with the Silica Eye Drop Product may be less significant to patients due to the greater convenience they offer, as well as the regimen providing doctors with better control over treatment.

Throughout the entire 15‐day period of the clinical study, the Silica Eye Drop Product showed acceptable clinical tolerability. There were no observable changes in the eye's structure compared to the control group. The trial did not identify any risks during its conduct or review of the results, either. However, minor observable changes in corneal and conjunctival fluorescein staining patterns were detected, although only in some subjects. These findings suggest that the Silica Eye Drop Product does not appear to have a toxic effect on surrounding tissues, even for an extended period of 15 days. This is also confirmed by another study on the effect of soluble silicic acid on ocular epithelial cells in vitro (Poluianova et al., 2024). Silicic acid is the degradation product of silica in the tear fluid and is likely present in the tissue surrounding the administration site, which could have an impact on the epithelial cells.

It was crucial to assess the pleasantness of using the Silica Eye Drop Product, as this factor impacts patient compliance and the continuation of drug usage. Existing studies have focused on the pleasantness (McMullin et al., 2021; Regnault et al., 2010) of using different eye drops, but our data is limited to healthy volunteers without any symptoms for medical therapy administration. The pleasantness score value of 4.9 ± 1.83 indicates that the use of the Silica Eye Drop Product falls within the neutral range, suggesting a balanced response without significant levels of either pleasantness or unpleasantness. This implies that the product is unlikely to cause substantial negative feedback from patients.

All our data demonstrate that using the Silica Eye Drop Platform has a favourable profile, demonstrating good tolerability in volunteers for up to 15 days. This suggests that IMD can be instilled long‐term, as our extended study showed no significant pathological changes in the eye structures. This is especially important for patients with chronic conditions, such as dry eye syndrome, glaucoma, or activation of iritis, when it is necessary to administer medications for a couple of months. The absence of pronounced side effects indicates a low risk of cumulative irritation or toxicity with repeated daily use.

In addition, the obtained tolerability data can be used in planning our future stages of drug development and clinical studies. In particular, it allows us to determine safe concentration ranges and frequency of application, which can contribute to a more efficient design of early‐phase clinical trials and reduce the risk of AEs. The results also create opportunities for further use of the Silica Eye Drop platform as a carrier for active ophthalmic drugs, such as antiglaucoma, anti‐inflammatory, or moisturizing agents. From our perspective, there are no limitations regarding the chemical nature of the medication, as the silica matrix can be adapted accordingly.

Currently, most studies are testing the tolerance of transport systems and the effectiveness of different medications at the same time, making comparison to our clinical trial more complicated (Goldberg et al., 2019; Gudmundsdottir et al., 2014; Sánchez‐González et al., 2024). Craig et al. (2010) demonstrated positive tolerance for their non‐encapsulated product, a liposomal spray, with 68% of the subjects preferring this spray over the control due to its comfort properties. It also demonstrated that its short‐term use on the eye's surface improved lipid layer grade and non‐invasive tear film stability. In this case, the application of liposomes was beneficial due to their physiological properties rather than a reduction in the number of applications.

There are several limitations in our study. Firstly, it is constrained by the comparatively limited sample size and only 15 days of observation, which restricts the ability to assess long‐term safety and potential efficacy in chronic use. Secondly, the challenges associated with self‐administration of the drug by patients warrant special attention. While the instillation was performed in a controlled environment by a study nurse or self‐administered by relatively young, healthy adult volunteers during the clinical study, patients with limited motor skills, elderly individuals, or those with visual impairments may struggle with accurate dosing and proper instillation technique in real‐world practice. It is also essential to evaluate the Silica Eye Drop Product within the context of existing ophthalmic delivery forms. Unlike traditional solutions and suspensions, the preparation is based on a silica‐silica composite, so assessing the risks linked to potential particle accumulation or their interaction with the tear film during chronic use is necessary. Finally, the overall pleasantness of using the product is moderate, as it is relatively dense and opaque.

Despite the limitations, our study represents the pioneering administration of silica composite formulations in the human eye. Additional clinical trials are necessary to evaluate the efficacy of utilizing silica microparticles containing encapsulated drugs as medication in specific patient cohorts. These trials would provide valuable insights into the overall impact and effectiveness of this drug delivery system, covering both anterior and posterior segment eye disease treatments.

In summary, the current article demonstrates that the topical ocular administration of Silica Eye Drops for at least 15 days, once daily, is safe and well tolerated by healthy adult subjects, with only mild to moderate ocular discomfort reported.
